# Long Arm Casting for Treatment of Trigger Finger in Children: Report of Three Cases

**Published:** 2012-03-05

**Authors:** SH Saeed Banadaky, B Baghianimoghadam

**Affiliations:** * Orthopedic Surgery Department, Shahid Sadoughi University of Medical Sciences, Yazd, Iran; ** Research consultant, Yazd, Iran

**Keywords:** trigger digit, infant, long arm casting

## Abstract

Trigger digit in children is rare. Triggering predominantly occurs in thumbs presenting a flexion deformity of interphalangeal joint of finger. In children, the disease usually presents with a remained finger in locked flexion, unlike the adults in whom triggering is more prevalent. The pathology of the disease includes locking of the tendon over A1 pulley. Some treatment modalities have been suggested to cure the trigger thumb, such as conservative therapy without any invasive approach, and surgery. To the best of our knowledge, there is no report about casting as a treatment method for trigger finger in children. Herein, we report three cases of patients with trigger finger, who were treated by using long arm casting.

## Introduction

Trigger digit is an uncommon condition in infants and children. Its estimated incidence rate is of 0.05% to 0.3% [**1**]. 2% of the upper limb congenital anomalies are trigger digits; although some authors believe that this is an acquired rather than a congenital anomaly [**2-4**]. The thumb is much more frequently affected than the other digits [**5,6**]. The disease presents with a flexion deformity of the interphalangeal joint of the finger. It is more commonly unilateral. Unlike adults, in children the most affected digits do not trigger but remain in locked flexion [**5-7**].

The pathology of the disease is the locking of the flexor tendon over A1 pulley, with changes in both the pulley and tendon. Reports about the natural history and the treatment approaches to trigger finger in children are limited. There is also sparse information about possible non-surgical treatment for trigger finger. To the best of our knowledge, there is no report about casting as an alternative treatment modality. Herein, we report three cases of patients with trigger finger, who were treated by using long arm casting.

## Cases presentation

The first patient was a 32-month-old boy who presented with triggering of his left ring and locked middle finger. His middle finger was operated and A1 pulley was released, but the triggering finger was not resolved successfully. So, we extended the incision along the finger, so that the longitudinal bulging of tendons, especially the flexor digitorum profundus (FDP), could be seen. Then reduction tenoplasty was conducted by using the technique described by Seradge [**8**]. Thereafter, a long arm cast (covering the metacarpophalangeal joints) was applied for tendon healing, for three weeks. We applied the long arm cast with Plaster of Paris and covered the fingers in neutral position and elbow in 90 degree angle. Hand webs were padded sufficiently to prevent the skin maceration and the elbow was covered by the cast to prevent the cast slippage. After three weeks, when we removed the cast, we found that not only the middle finger was cured, but also the triggering of the ring finger had been resolved.

The second patient was a 26-month-old boy with a right middle finger triggering. He was previously operated on and the A1 pulley had been released, but the symptoms continued. Having considered the first experience, we tried a long arm cast for three weeks. After that period the symptoms disappeared.

The third patient was another 20-month-old boy who presented with a right middle finger triggering. A similar approach was done and long arm cast was applied for a three-week period. The symptoms were completely resolved after that period. 

We followed up the patients for an average period of 38 months and found that they fully recovered. The second and third patients had only some intermittent catching in the early follow-up visits, but no more interventional treatments were needed.

## Discussion

Various possible modalities for treating trigger thumbs have been proposed in children because triggering is more frequent in thumb than in the other fingers. Spontaneous resolving was reported in patients with mild symptoms or in young ages (12 months old or less) [**9,10**]. Baek et al. reported that more than 60% of the affected patients will be symptom free without a treatment, after a suitable period of follow up and the remaining patients will show some features of improvement [**11**]. Most of the similar reports are about trigger thumb, while trigger digit cases have sparsely been reported.

The report of using splinting in adults with trigger fingers indicates possible benefits; however, those results cannot be extrapolated in children because many of such adult patients have systemic diseases, including inflammatory arthritis [**12**]. Lee et al. reported that the extension splint could lead to cure or improvement in up to 71% of their patients aged 0-4 years.

About one third of their studied patients, for whom elective surgery was suggested, did not improve [**13**]. Although we cannot extrapolate our results by treating only three patients, we used only three weeks casting for our patients, which is shorter and more acceptable compared with the mean 11.7 weeks splinting, done by Lee and colleagues. Long arm casting might lead to more immobilization compared with splinting; hence shorter period of treatment was needed.

Surgery has been recommended for persistent cases that are not responsive to noninvasive treatments; results of long term follow-up after elective surgery was good [**14**].

McAdams et al. reported the results of operation on 30 triggering thumbs [**15**]. After an average 15 years follow-up, they did not find any recurrence or functional loss. However, some complications, such as loss of interphalangeal motion and metacarpophalangeal hyper extension, were major concerns. Having considered that surgery is not urgent in trigger finger and can be done electively, so as to avoid the above mentioned complications, our proposed method for a three-week long arm casting can be a good treatment modality. Surgeries can be reserved for persistent cases which do not respond to the other modalities, although our acceptable long-term 38-months follow-up could suggest that this method of treatment might be effective in other patients.

## Conclusions

Immobilization for 3 weeks long arm casting might be a new alternative treatment for trigger finger. It is possible that hand rest could resolve tendon catching in fibro-osseous tunnel. Although treating three patients cannot supply enough evidences to suggest casting as the first choice and larger clinical trials with control group is needed, 3-week long arm casting can be an alternative suggestion before any decision making for invasive procedures.

## References

[1] Ger  E, Kupcha  P, Ger  D (1991). The management of trigger thumb in children. J Hand Surg [Am].

[2] Rodgers  WB, Waters  PM (1994). Incidence of trigger digits in newborns. J Hand Surg [Am].

[3] Mulpruek  P, Prichasuk  S, Orapin  S (1998). Trigger finger in children.. J Pediatr Orthop.

[4] Slakey  JB, Hennrikus  WL (1996). Acquired thumb flexion contracture in children: congenital trigger thumb. J Bone Joint Surg [Br].

[5] Fahey  JJ, Bollinger  JA (1954). Trigger-finger in adults and children. J Bone Joint Surg Am.

[6] Wood  VE, Sicilia  M (1992). Congenital trigger digit. Clin Orthop Relat Res.

[7] McCarroll  HR Jr. (1985). Congenital flexion deformities of the thumb. Hand Clin.

[8] Seradge  H, Kleinert  HE (1981). Reduction flexor tenoplasty. Treatment of stenosing flexortenosynovitis distal to the first pulley. J Hand Surg Am.

[9] Ger  E, Kupcha  P, Ger  D (1991). The management of trigger thumb in children. J Hand Surg [Am].

[10] Moon  WN, Suh  SW, Kim  IC (2001). Trigger digits in children. J Hand Surg [Br].

[11] Baek  GH, Kim  JH, Chung  MS (2008). The natural history of pediatric trigger thumb. J Bone Joint Surg Am.

[12] Colbourn  J, Heath  N, Manary  S (2008). Effectiveness of splinting for the treatment of trigger finger. J Hand Ther.

[13] Lee  ZL, Chang  CH, Yang  WY (2006). Extension splint for trigger thumb in children. Pediatr Orthop.

[14] van den Borne MP, Custers FJ, van der Aa JP (2000). Trigger thumb' in 38 children: good short-term and long-term results from surgery. Ned Tijdschr Geneeskd.

[15] McAdams TR, Moneim  MS, Omer  GE Jr (2002). Long-term follow-up of surgical release of the A(1) pulley in childhood trigger thumb. J Pediatr Orthop.

